# Geographic Range Size Predicts Butterfly Species' Tolerance to Heavy Metals More Than Evolutionary History With Toxic Larval Diets

**DOI:** 10.1111/eva.70114

**Published:** 2025-05-26

**Authors:** Ashley L. Darst, Lindsey R. Kemmerling, Molly Tilsen, J. Alexander Eilts, Emilie C. Snell‐Rood

**Affiliations:** ^1^ Department of Ecology, Evolution and Behavior University of Minnesota Twin Cities Minnesota USA; ^2^ Department of Integrative Biology Michigan State University East Lansing Michigan USA; ^3^ W.K. Kellogg Biological Station Michigan State University Hickory Corners Michigan USA; ^4^ Water Resources Science University of Minnesota Twin Cities Minnesota USA; ^5^ College of Biological Sciences Conservatory University of Minnesota Twin Cities Minnesota USA

**Keywords:** Ames test, arsenic, cadmium, heavy metals, lead, Lepidoptera, manganese, preadaptation, range size, urban ecology

## Abstract

Some organisms appear to thrive in contaminated environments, while others are more sensitive, though the causes of this variation are unclear. The toxin coevolution hypothesis posits that an evolutionary history with natural toxins preadapts species to deal with novel toxins, while the range‐size‐tolerance hypothesis posits that a larger geographic range selects for broader tolerance to stressors. Butterflies are a prime system to investigate these hypotheses because they are diverse, feed on a range of larval host plants that vary in defensive compounds, and many are found in polluted environments. We ask how these hypotheses explain varying tolerance to heavy metal pollution, measured here as loads of four heavy metals along an urban gradient of metal exposure. We compared 26 butterfly species that vary in their evolutionary history with mutagenic plant defensive chemicals as well as their geographic range size. We built a dataset of plant mutagenicity synthesizing 40 years of standardized mutagenicity screening in plants, including 502 plant species of 103 families within 37 orders. We used this dataset, coupled with butterfly host records, to estimate evolutionary history with mutagens. We found that butterfly species with larger ranges tolerated significantly greater concentrations of lead, arsenic, and cadmium in their tissues. Additionally, species with a history of feeding on relatively more mutagenic host plant families tolerated greater maximum lead concentrations in their thoracic tissue. This research provides additional support for the growing observation that small‐ranged species are more vulnerable to environmental change, in this case, metal pollution. In addition, an evolutionary history with mutagenic host plants may provide some additional resilience, although less than geographic range size. In addition, our dataset on comparative plant mutagenicity will facilitate future research on plant‐herbivore coevolution, in fields such as chemical, community, and urban ecology.

## 1 Introduction

Over recent centuries, humans have generated a large amount of pollution, from heavy metals and plastics to acid rain and radiation. Although pollutants have caused the decline of many species, some organisms are able to tolerate high levels of pollutants (Bothe and Slomka 2017; Janssens et al. 2009; Nanda et al. 2019). This is perplexing because many human pollutants are, from an evolutionary perspective, novel chemicals (Wackett 2024) or novel combinations of chemicals (e.g., “chemical cocktails” Kaushal et al. 2020). There are instances of species evolving specific adaptations to cope with elevated pollutants (van Ooik and Rantala 2010), but the question still remains: why are some organisms able to cope with a novel human pollutant in the first place? Recent research has focused on pre‐existing generalized stress mechanisms that allow tolerance of novel toxins (Li et al. 2007; Snell‐Rood et al. 2018). However, other mechanisms exist, including broad tolerance to environmental variation. In this research, we test two hypotheses to explain why species vary in their ability to tolerate pollutant exposure.

First, we focus on the toxin coevolution hypothesis—that an evolutionary history with plant chemical defensives should bias some species to deal with novel toxins (Li et al. 2007). Toxins occur naturally throughout the biological world, such as defensive chemicals against consumers (Mason and Singer 2015; Negin and Jander 2023; Nishida 2014) or venoms used by snakes or scorpions to immobilize prey (Calvete 2017; Jared et al. 2021; Lachance et al. 2012). Co‐evolutionary interactions between species often result in the evolution of tolerance of these defensive or offensive chemicals, such as woodrats being immune to rattlesnake venom (Perez et al. 1978). The pathways adapted for dealing with natural toxins may be evolutionarily co‐opted for dealing with novel toxins (Bernhardt 2006; Efferth and Volm 2017; Li et al. 2007). For instance, in *Pieris* butterflies (Lepidoptera: Pieridae), upregulated general antioxidant stress pathways may have allowed the tolerance of novel plant toxins (Sikkink et al. 2020), potentially facilitating shifts to new diets and the subsequent evolution of more specific enzymes to break down these chemicals (Wheat et al. 2007). The physiological mechanisms that allow tolerance of historical toxins are often at least somewhat “general” in that they confer resistance to other toxins (Sikkink et al. 2020; Snell‐Rood et al. 2018).

An additional hypothesis, what we term the range‐size‐tolerance hypothesis, posits that tolerance to novel toxins should increase with range size due to broad environmental tolerance. This hypothesis stems from the observation that across many species, there are strong correlations between a species' range size and their breadth of tolerance to physiological stressors, especially temperature (e.g., Calosi et al. 2008; Gaston and Spicer 2001; Saetersdal and Birks 1997, rev. Bozinovic et al. 2011). In addition, organisms that thrive in urban environments also tend to be those with larger ranges and broader environmental tolerance (Bonier et al. 2007). The direction of causality likely goes both ways—a broad tolerance facilitates range expansion, which further selects for broad tolerance through increased environmental variation (Snell‐Rood and Steck 2019). Although the link between range size and tolerance has been best studied with respect to temperature stress, it has been argued that range size should be more generally correlated with tolerance to multiple stressors (Bozinovic et al. 2011). For instance, butterflies that perform well in urban and human‐dominated environments tend to have larger ranges (Clark et al. 2007; Jahner et al. 2011; Sunde et al. 2023), likely because species with large ranges have adapted to a broad thermal tolerance and host breadth (Brändle et al. 2002; Garcia‐Barros and Romo Benito 2010; Hausharter et al. 2023).

Here, we use anthropogenic heavy metal pollution to contrast the toxin coevolution and the range‐size‐tolerance hypotheses as explanations of why organisms vary in their tolerance to pollutants. Heavy metals are elements with a high atomic weight and density that are often toxic to life (Balali‐Mood et al. 2021; Mason et al. 2014; Tchounwou et al. 2012). Although they are naturally occurring in the Earth's crust, anthropogenic activities such as mining, pest control, and smelting have increased the relative availability of heavy metals in the environment (Briffa et al. 2020; Tchounwou et al. 2012), often in novel combinations (Kaushal et al. 2020). Despite the toxicity of heavy metals, some organisms can tolerate heavy metal exposure (Bothe and Slomka 2017; Pollard et al. 2014) and cope with elevated levels in anthropogenic environments—for instance, through upregulation of metal‐processing genes (Sunitha et al. 2013). Heavy metals directly or indirectly create free radicals and DNA damage (Balali‐Mood et al. 2021; Bertin and Averbeck 2006), similar to mutagenic chemicals more broadly (Phaniendra et al. 2015). For example, aristolochic acids, which are found in plants in the family Aristolochiaceae, are potent mutagens in bacteria, *Drosophila*, and humans (Chen 2007). At the same time, the entire Troidini tribe of swallowtail butterflies has been coevolving with these toxins for millions of years, some even coating their eggs with them as a defense (Nishida and Fukami 1989; Sime et al. 2000). Is it possible that coevolution with plant mutagens could preadapt some butterflies to cope with heavy metal stress?

We focus on butterflies as a model system to test range size and history with mutagenic chemicals as preadaptations to cope with heavy metal pollution. The caterpillars of most butterfly species feed on only one or two plant families (i.e., host plants), and across the 19,000+ species of butterflies in the world, there are hundreds of host plant shifts across different plant families (Ehrlich and Raven 1964; Scott 1992). Many of these host plants produce chemical defenses that also happen to be mutagenic, including the aristolochic acids mentioned above, but also pyrrolizidine alkaloids in the family Asteraceae (Chen et al. 2010; Clark 1960; Dreger et al. 2009; Fu et al. 2004), and cycasins in cycads (Morgan and Hoffmann 1983). In contrast, some other plant groups, such as grasses, tend to have structural rather than chemical defenses and are rarely mutagenic (Currie and Perry 2007; Dantas et al. 2020). The chemistry of plants represents a vast undescribed space; indeed, some studies suggest that only 1% of such chemical diversity has been described (Domingo‐Fernández et al. 2023; Farnsworth 1988). In this work, we first develop a database to estimate the likelihood that a given plant family produces mutagenic defensive chemicals. We rely on the ubiquity of the “Ames test,” a common screen for mutagenicity of a chemical or extract (Ames 1983; Ames et al. 1973; Mortelmans and Zeiger 2000), compiling data from hundreds of studies on the mutagenicity of plant extracts (building on previous literature reviews—Dantas et al. 2020). We then align these data with information on the host plants of a given butterfly species (Scott 1992) to estimate their evolutionary history with mutagens and contrast this predictor with a species' geographic range size.

Studying pollutant tolerance across species is challenging due to the limits of raising multiple species in controlled conditions that vary in metal exposure. Thus, we develop field‐based methods to understand variation in metal tolerance across species. We collected 26 species of butterflies over a gradient of heavy metal pollution in the Twin Cities (Minneapolis and St. Paul, Minnesota, USA). We use several measures of a species' distribution of metal tissue burden (maximum and mean metal concentration) as a proxy of tolerance to metal exposure. We focus on four metals with known mutagenicity and with levels of concern in the Twin Cities area: lead, arsenic, cadmium, and manganese. We use body metal concentration of these metals to test the prediction that species with an evolutionary history with mutagenic plant families and/or a broader geographic range show higher levels of heavy metal tolerance along an anthropogenic gradient of metal pollution.

## 2 Methods

### 2.1 Study Area

We make use of an urban‐to‐rural gradient of heavy metal exposure across the Twin Cities, which represent two urban cores with a typical history of urban heavy metal pollution (Mielke 1993; Mielke and Adams 1989; Mielke et al. 1984) from past and current industry (e.g., manufacturing of lead bullets), chemical production (e.g., arsenic based pesticides), use of metal‐containing products (e.g., lead paint and lead gasoline), and the general wear and tear of metal‐containing human products (e.g., tires, brake pads, pipes). Heavy metal research is an ongoing focus of the Minneapolis‐St. Paul Urban Long‐Term Ecological Research Program, which is documenting spatial and temporal patterns of metal pollution in the two cities.

**FIGURE 1:**
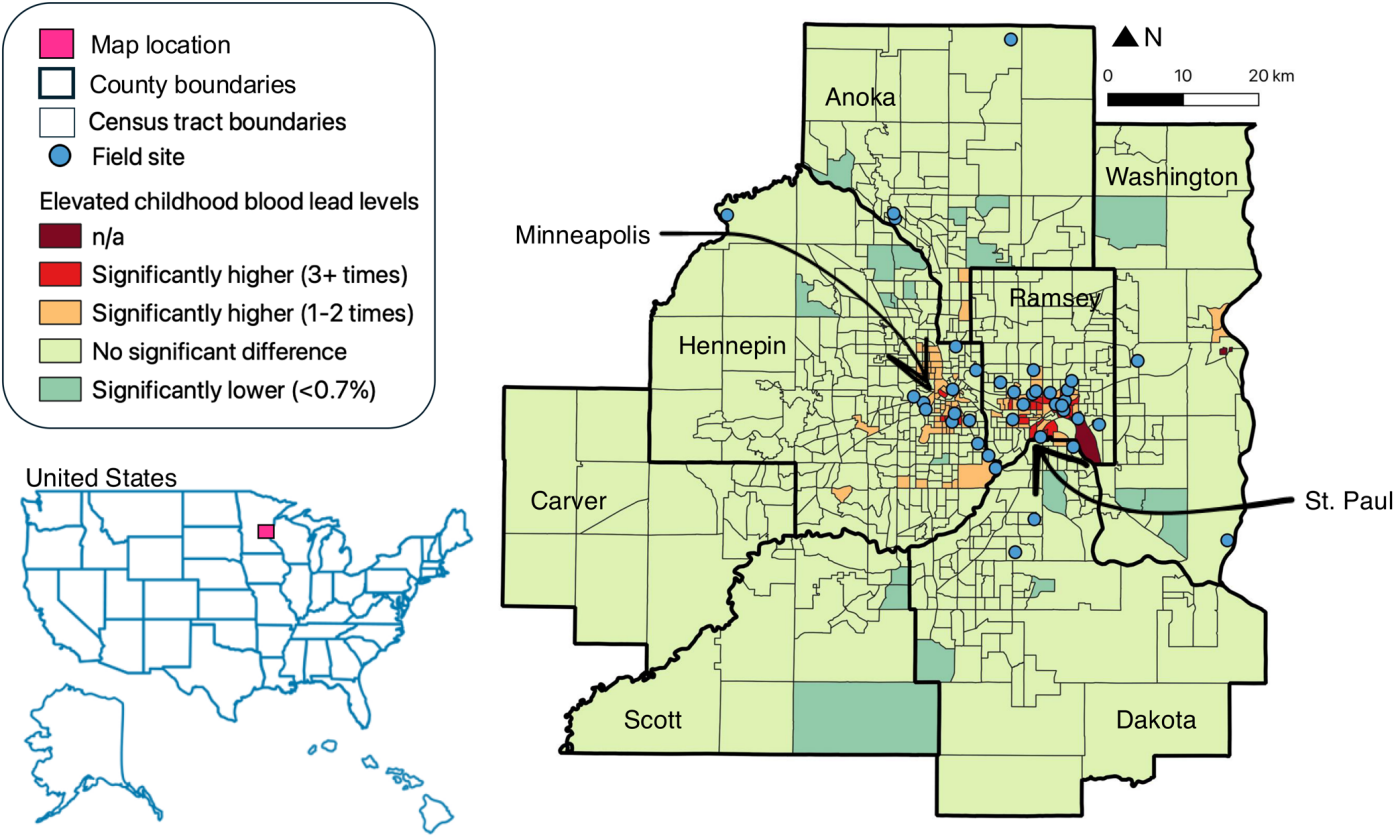
Field sites across the Minnesota Twin Cities seven‐county metropolitan region. Field sites are denoted with blue points. Teal census tracts have significantly less elevated childhood blood lead levels than Minnesota overall. Light green census districts do not have significantly different elevated childhood blood lead levels than Minnesota overall. Orange census districts have significantly higher elevated childhood blood lead levels by 1–2 times than Minnesota overall. Red census districts have significantly higher elevated childhood blood lead levels by 3 or more times than Minnesota overall. Maroon districts do not have childhood blood lead data. County names are included, and black arrows point to the city cores of Minneapolis and St. Paul. Map created by Lindsey Kemmerling with data from the Minnesota Department of Health.

Butterflies were collected from 34 sites (parks, gardens, and yards) across the Minnesota Twin Cities metropolitan region between June 2022 and August 2023 (Figure 1; *N* = 689 from 2022, *N* = 41 from 2023). To collect samples across a gradient of heavy metal pollution, sites were selected using data from the Minnesota Department of Health on elevated childhood blood lead levels (https://mndatamaps.web.health.state.mn.us/interactive/leadtract.html). This database is a rough proxy for soil lead concentrations across the Twin Cities (Jelinski unpublished; Mielke and Adams 1989). We sampled from a roughly equal distribution of sites from unelevated childhood blood lead levels (*N* = 13), slightly elevated levels (1–2× above average, *N* = 12), and highly elevated levels (3× and above, *N* = 10) relative to Minnesota overall. We additionally collected soil and plant samples from our sites for a separate study on ecological correlates of elevated metal levels in butterflies (Kemmerling, Darst et al. 2025; Kemmerling, Snell‐Rood et al. 2025). Informed consent was not applicable.

### 2.2 Butterfly Sampling

We focused on butterfly species where we captured at least 10 individuals (*N* = 26). Across all butterflies, each species was sampled from on average 10 different sites (range = 3–28 sites, Figure S1). By collecting species at as many sites as possible, we expected to sample individuals from a range of larval metal exposures. For most species in the study, average daily dispersal distances range from 23 to 660 m (Stevens et al. 2010), with larger butterflies typically dispersing farther than smaller butterflies (Sekar 2012).

We aimed to collect common species that could be found across the whole heavy metal pollution gradient, maximizing the number of different larval host species (and independent host shifts). For several lineages on a given larval host, we have a range of replicate species feeding on that host, which allows statistical power to tease apart the effects of evolutionary histories with mutagens (determined by larval host plant family) and geographic range size. This is particularly true for grass‐feeding skippers (Hesperiinae), grass‐feeding satyrs (Satyrinae), and legume‐feeding pierids (Colianae). Note that due to difficulties with identification and the debate surrounding the classification of the *tharos* group species during the time of this study (Zhang et al. 2022), we considered all potential *Phyciodes tharos* or *Phyciodes selenis* individuals as belonging to one species (but see Wingert et al. 2024). Both species feed on asters and have similar range sizes, so this decision should not meaningfully impact our results and conservatively reduces the number of species in the study.

### 2.3 Heavy Metal Analysis

To choose focal metals for analysis, we used soil data from the Twin Cities (Kemmerling, Snell‐Rood et al. 2025; Jelinski et al. unpubl.) coupled with toxicity values for those metals (Minnesota Pollution Control Agency 2024). We chose metals where the 90th percentile of soil metal concentrations was above the threshold of concern for toxicity. Previous research on butterflies in this region showed that soil levels of metals were more tightly correlated to body metal content than host plants (Kemmerling, Darst et al. 2025; Kemmerling, Snell‐Rood et al. 2025). For sites across the area, soil levels (at the 90th percentile) for lead, arsenic, cadmium, and manganese were elevated by 25%, 57%, 87%, and 7% above levels of concern. All of these metals have known mutagenicity effects (lead: Ostoich et al. 2020; cadmium: Filipic et al. 2006; arsenic: Tchounwou et al. 2004; manganese: Gerber et al. 2002).

We focused on metal concentrations in the butterfly thorax as a proxy for larval metal exposure. Butterfly thorax is primarily composed of flight muscle (Marden 2000; Smith 1965), and most butterfly protein is derived from the larval diet (O'Brien et al. 2005, 2002). For each individual, the thorax was separated from the wings, head, and abdomen with forceps and dried at 70°C for at least 24 h.

Metal concentrations of butterfly tissue were measured with Inductively‐Coupled Plasma Mass Spectroscopy at Northwestern University's Quantitative Bio‐element Imaging Center. Thoraxes were weighed in batches of 15 or less and designated as large (> 15 mg) or small (< 15 mg). They were acid digested in a 4:1 ratio of HNO_3_ to H_2_O_2_ for 3 h, with 625 μL for large samples and 312.5 μL for small samples. Samples were then diluted with Millipore water—10 mL total for large samples and 5 mL total for small samples—and mixed. Large samples were analyzed with 4× auto‐dilution and small samples were analyzed with 1× auto‐dilution. Each butterfly sample was run three times and technical replicates were averaged.

For our metals of interest, a number of samples were below the limit of detection (LOD), which varied across metals and three separate ICP‐MS runs (LODs: lead—0.002, 0.002, 0.002 ppb, arsenic—0.003, 0.006, 0.01 ppb, cadmium—0.001, 0.001, 0.002 ppb, and manganese—0.003, 0.006, 0.01 ppb). Our analyses censored these data as explained in the statistics section below (0 samples of 733 for manganese, 2 for arsenic, 93 for cadmium and 155 for lead). In our study, smaller species were more likely to return samples below the limit of detection for lead (Figure S2), suggesting these “below LOD samples” were primarily determined by the amount of available sample and the limits of the machine, rather than the biological reality of truly low values.

### 2.4 Host Plant Mutagenicity

We built a database of plant mutagenicity using published Ames test data (Ames et al. 1973; Mortelmans and Zeiger 2000). The details of this database can be found in the Supporting Information (Appendix S1) and Darst et al. (2025). Briefly, in an Ames test, mutated bacteria are exposed to a potential mutagen relative to control conditions (Ames 1983; Ames et al. 1973, 1987). The number of revertant bacteria is compared between the treatments. An extract or compound is considered mutagenic if there are at least twice as many revertant bacteria in the potential mutagen treatment. We used standard search terms in the search engine “Web of Science” to find Ames test data from extracts of plant tissue (primarily leaves). We manually combed through over 2000 references to find papers that ran Ames tests on plant tissue extracts. In total, our database compiled data from 163 studies (from 1983 to 2023), including 502 species of 103 plant families within 37 plant orders. We used a categorical response variable (“was the extract mutagenic?” – yes or no) which facilitated combining results across studies. However, key aspects of the methodology varied across studies (e.g., chemical used during the extraction, bacterial strain used), which we control for in our final measures of mutagenicity (see details in Supporting Information). For each plant family with at least eight Ames tests, we calculated the family mutagenicity score as the proportion of tests that detected mutagenicity, using a metric that corrected for variation in methodology. For plant families with fewer than eight tests, we used the value as calculated at the plant order level. Butterfly host plant records were taken from Scott (1992). To assess evolutionary history of a butterfly species with mutagenic host plants, we calculated a mutagenicity score for each butterfly species, weighted by their percent usage of host plants from different plant families. For instance, if 80% of a species hosts were in Brassicaceae and 20% in Capparaceae, we calculated the mutagenicity score as 0.8 × (mutagenicity of Brassicaceae) + 0.2 × (mutagenicity of Capparaceae).

### 2.5 Species Range Estimates

We calculated the area of species' North American ranges using published integrated North American ranges maps (Grames et al. 2024). In short, Grames et al. extracted ranges from field guides created by experts and calculated habitat suitability with MaxEnt models using Global Biodiversity Information Facility (GBIF) records. None of our 26 species' ranges were fit with pseudo‐presence points. We uploaded the integrated range maps for the species in this study as raster objects in R v4.4.1 using the *raster* package v3.6‐30 (Hijmans 2024; R Team 2022). We excluded cells with predicted habitat suitability < 0.2 (Grames et al. 2024). We calculated the area of the remaining cells using the area function in the *raster* package. We added the cell areas together to get the total area of the range in km^2^. As we did not distinguish between *Phyciodes selenis* and *Phyciodes tharos* , we calculated the average of their ranges, which were similar sizes ( *Phyciodes selenis* = 8,717,507 km^2^, *Phyciodes tharos* = 7,416,403 km^2^).

### 2.6 Statistical Analysis

We were first interested in whether butterfly species showed differences in metal concentrations, warranting a comparative analysis of such values. We tested for variation in metal concentrations across species specimens using a generalized regression (normal distribution) that accounted for samples below the limit of detection using a maximum likelihood approach in JMP v17 (SAS Institute). As a proxy for a species' metal tolerance, we used both the maximum metal concentration observed for that species in addition to the “predicted” mean value from the generalized regression model that accounted for censored individuals below the limit of detection. To censor the data, values below the limit of detection (or those returned as negative values) were scored as “0” and the average limit of detection for the metal (see above) coded into the variable properties in JMP. Because we focused metal analyses on log_10_‐transformed concentrations, we first added one to each metal value to ensure all log‐transformed values were above zero to facilitate this approach.

To test the hypotheses that a butterfly species range size and/or evolutionary history with plant mutagens predicted their metal concentrations, we used phylogenetic generalized least squares regression (PGLS), implemented in R v4.4.1 using the *caper* package v. 1.0.3 (Orme et al. 2012; R Team 2022). For each metal, we fitted separate models with log maximum metal concentration or log predicted mean metal concentration as a function of range size and host plant mutagenicity (as noted above, log values were log_10_ (metal value +1) to facilitate censoring of values below the limit of detection). Both range size and host plant mutagenicity were scaled and centered to directly compare the relative effect of each variable and to improve model fit. We used phylogenetic relationships reported in Earl et al. (2021) for these analyses. As one species was absent from this phylogeny ( *Polites peckius* ), we used its sister species ( *Polites themistocles* ), which was present in the phylogeny, as an indicator of where it should fall in the tree (Zhang et al. 2021).

## 3 Results

### 3.1 Variation Across Butterfly Species in Traits

Butterfly species showed significant variation in average tissue concentration for the four focal metals, with “species” explaining the most variation in levels of cadmium, followed by manganese, lead, and arsenic (Table 1, Figure S3).

**TABLE 1:** Variation in species in metal concentrations. Shown are results of generalized regression on 730 butterfly samples testing for differences across 26 butterfly species in metal concentrations. This analysis used censored data that accounted for the average limit of detection for each model. We tested for species differences prior to running a comparative analysis (Table 2).

Metal	Wald *X* ^2^	*R* ^2^	*p*
Manganese	1460	0.468	< 0.0001
Arsenic	1434.7	0.267	< 0.0001
Cadmium	1216	0.507	< 0.0001
Lead	1009.2	0.309	< 0.0001

The range sizes of species (restricted to North America) varied from a minimum in *Satyrodes eurydice* (3,151,448 km^2^; east of the Rocky Mountains to the Atlantic Ocean and from New York, US to Winnipeg, CA) to a maximum in *Colias eurytheme* (11,660,266 km^2^; throughout Canada, the United States, and Mexico).

### 3.2 Plant Mutagenicity Data

**FIGURE 2:**
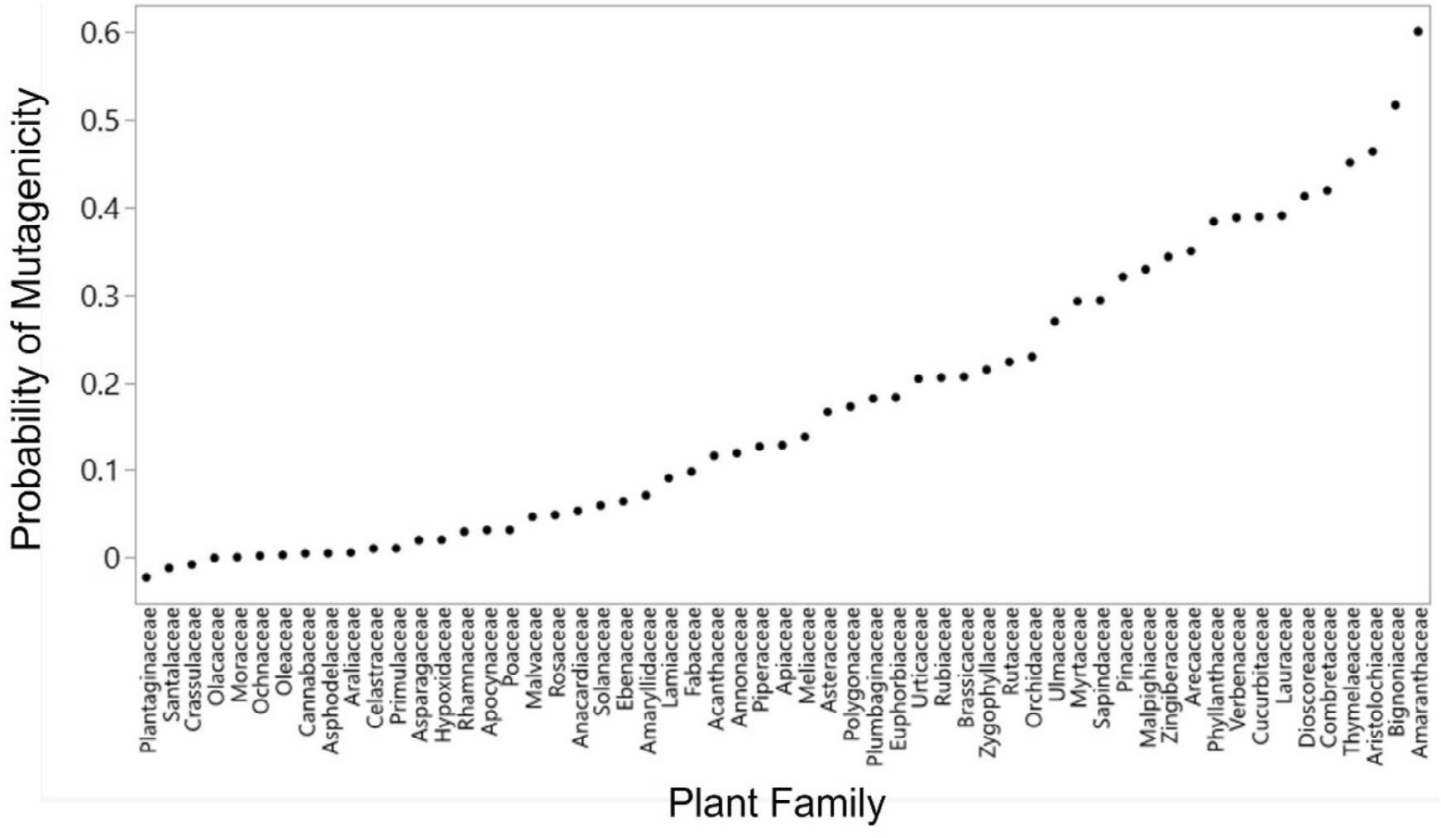
Variation across plant families in the likelihood a species is mutagenic. Based on Ames test data compiled across the literature (Supporting Information), this graph shows the probability that a given Ames test on a plant extract shows up as positive (mutagenic). These values correct for variation across studies in experimental details that affect the probability of a positive test (see Supporting Information) and should be treated as relative, not absolute, values. These values are limited to families where at least eight tests were present in our full dataset.

We compiled data from 163 studies that used the Ames test to measure the mutagenicity of extracts from diverse plants (Darst et al. 2025). Controlling for variation in methodology across 1610 Ames tests, there is a significant effect of plant family on the probability that an extract is mutagenic (see Supporting Information, *F*_56,283_ = 1.46, *p* = 0.024 for 56 plant families with at least 8 replicates). Of plant families commonly used as host plants of butterflies, some (e.g., Aristolochiaceae, Brassicaceae, Asteraceae) have a relatively greater chance of containing mutagenic chemicals than others (e.g., Cannabaceae, Apocynaceae, Poaceae; Figure 2).

### 3.3 Predictors of Butterfly Species Variation in Metal Concentrations

**FIGURE 3:**
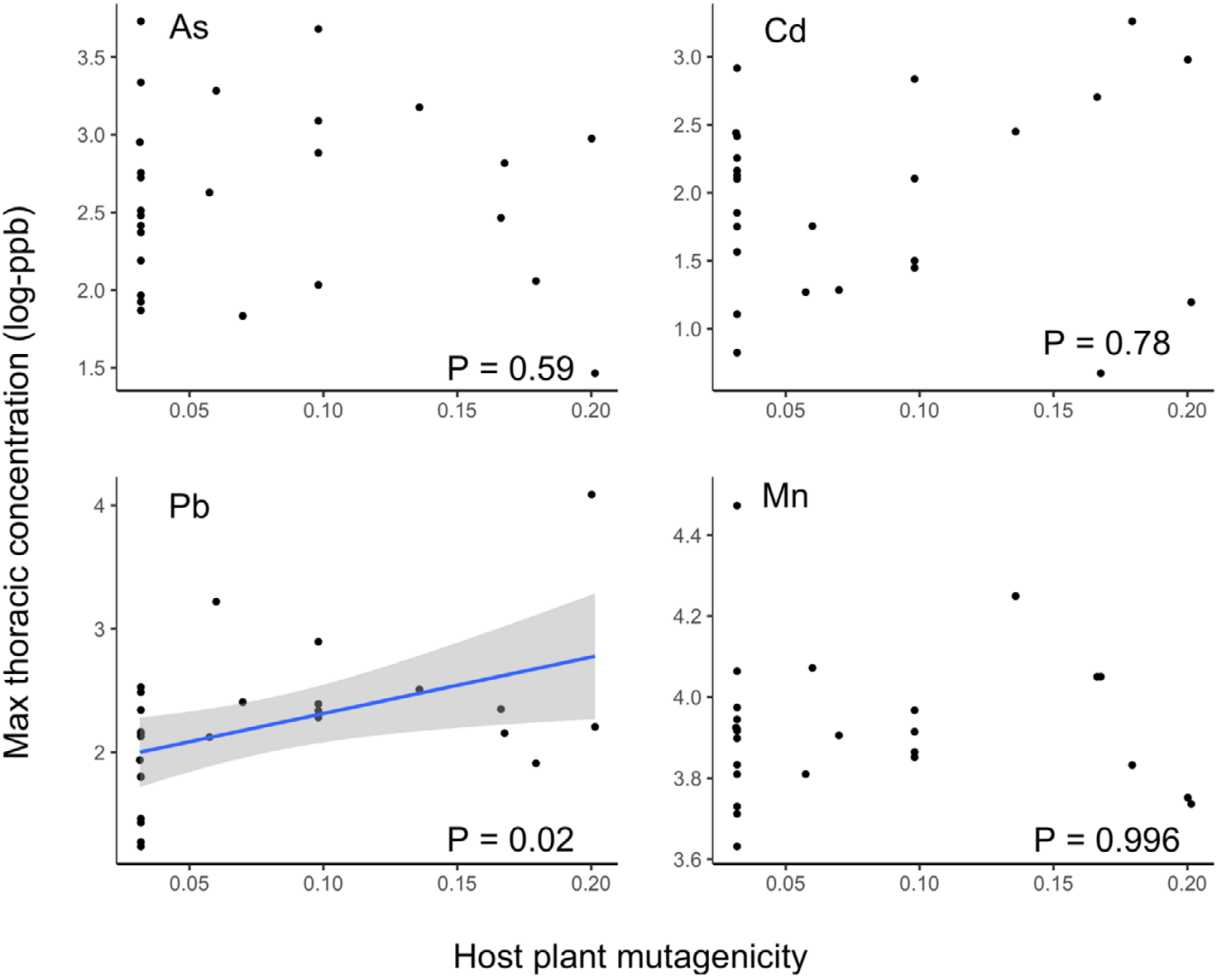
Variation across species in plant mutagenicity and maximum thoracic metal concentration. Shown are plots of each species mutagenicity index (*x*‐axis) and the maximum concentration of Arsenic (As), Cadmium (Cd), Lead (Pb), and Manganese (Mn) observed in field‐collected individuals. Metal concentrations are log‐transformed (after the addition of 1 to bound at 0, see Section 2). *p* Values are corrected for phylogenetic relationships and range size (see Table 2). Regression lines are shown only for significant relationships, and the shaded interval represents the 95% confidence interval.

**FIGURE 4:**
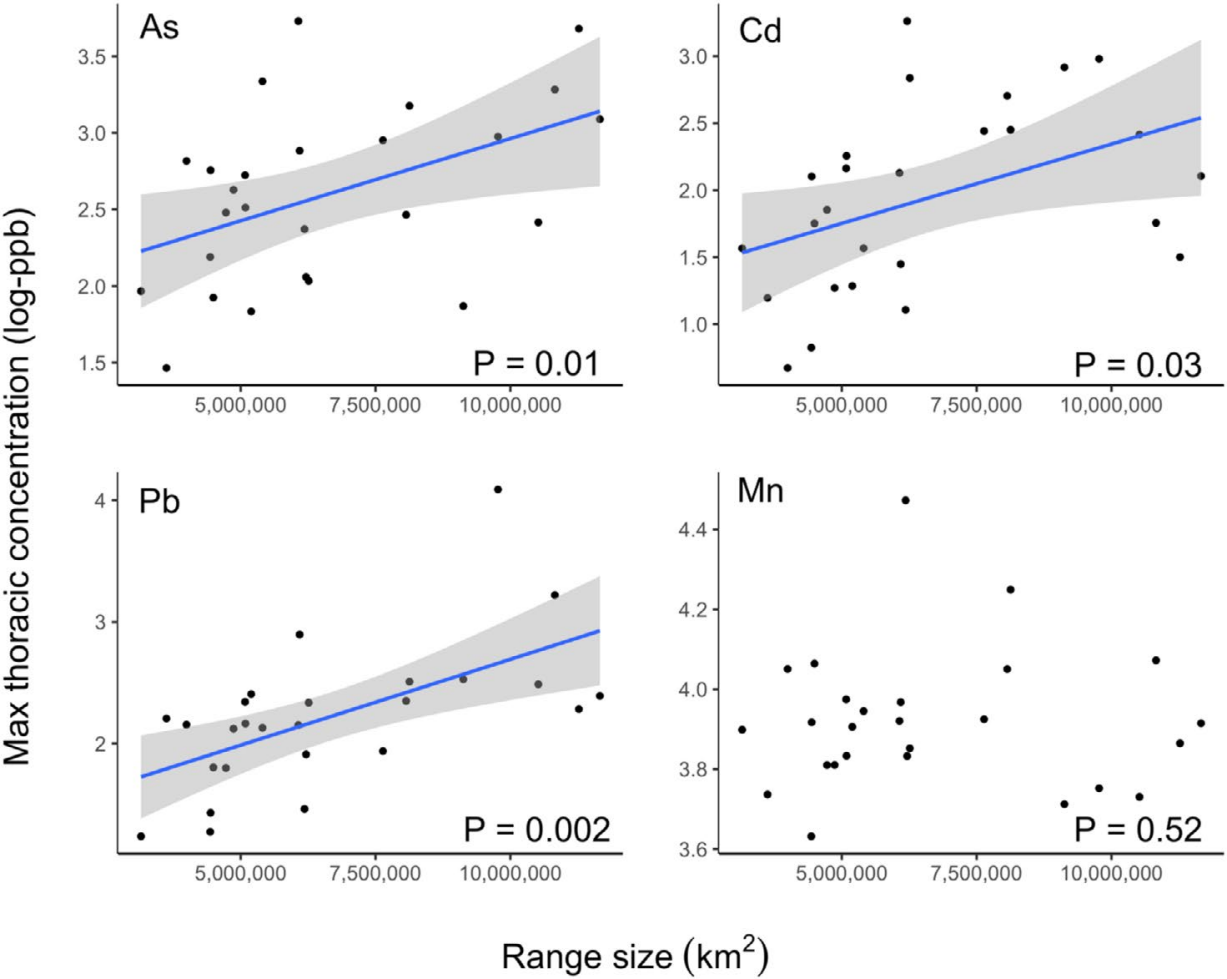
Variation across species in range size and maximum thoracic metal concentration. Shown are plots of each species range size (*x*‐axis) and the maximum concentration of Arsenic (As), Cadmium (Cd), Lead (Pb), and Manganese (Mn) observed in field‐collected individuals. Metal concentrations are log‐transformed (after the addition of 1 to bound the distribution to zero, see Section 2). *p* Values are corrected for phylogenetic relationships and mutagenicity index (see Table 2). Regression lines are shown only for significant relationships, and the shaded interval represents the 95% confidence interval.

A butterfly species' range size was significantly positively correlated with maximum and predicted mean concentrations of lead, arsenic, and cadmium when controlling for phylogenetic relationships (Table 2, Figures 3 and 4). A butterfly species' evolutionary history with mutagenic host plants was significantly positively related to concentrations of only one metal measure—maximum lead content (Table 2, Figure 3). Across models, predictor variables accounted for more variation across species in concentrations of lead, relative to arsenic and cadmium (Table 2). There were no significant predictors of variation across species in manganese concentrations (Table 2, Figures 3 and 4).

**TABLE 2:** Results of phylogenetically controlled least squares regression. We used PGLS to test for the importance of a species range size and evolutionary history with mutagenic host plants on the species' distribution of heavy metal concentrations. We used both maximum metal concentration and a predicted mean metal value that corrected for samples that fell below the limit of detection to capture the species variation. *R* ^2^ is the adjusted *R* ^2^.

		Dependent variable (metal)							
		Max (Mn)	Predicted (Mn)	Max (As)	Predicted (As)	Max (Cd)	Predicted (Cd)	Max (Pb)	Predicted (Pb)
Independent variables	Range size	*t* = 0.65	*t* = 1.05	*t* = 2.80	*t* = 3.08	*t* = 2.36	*t* = 2.95	*t* = 3.60	*t* = 2.68
		*p* = 0.52	*p* = 0.30	*p* = 0.01	*p* = 0.005	*p* = 0.027	*p* = 0.007	*p* = 0.002	*p* = 0.01
		Estimate = 0.02	Estimate = 0.02	Estimate = 0.30	Estimate = 0.14	Estimate = 0.31	Estimate = 0.29	Estimate = 0.31	Estimate = 0.22
	Mutagenicity	*t* = −0.005	*t* = 0.75	*t* = −0.54	*t* = −0.04	*t* = 0.29	*t* = 1.37	*t* = 2.51	*t* = 1.64
		*p* = 0.996	*p* = 0.46	*p* = 0.59	*p* = 0.97	*p* = 0.78	*p* = 0.18	*p* = 0.02	*p* = 0.12
		Estimate = −0.0002	Estimate = 0.02	Estimate = −0.06	Estimate = −0.002	Estimate = 0.04	Estimate = 0.16	Estimate = 0.22	Estimate = 0.14
Full model		*F*_2,23_ = 0.21	*F*_2,23_ = 0.76	*F*_2,23_ = 4.08	*F*_2,23_ = 4.79	*F*_2,23_ = 2.94	*F*_2,23_ = 4.85	*F*_2,23_ = 10.75	*F*_2,23_ = 5.46
		*p* = 0.81	*p* = 0.48	*p* = 0.03	*p* = 0.02	*p* = 0.07	*p* = 0.02	*p* = 0.0005	*p* = 0.01
		*R* ^2^ = −0.07	*R* ^2^ = −0.02	*R* ^2^ = 0.20	*R* ^2^ = 0.23	*R* ^2^ = 0.13	*R* ^2^ = 0.24	*R* ^2^ = 0.44	*R* ^2^ = 0.26
		*λ* = 0.546	*λ* = 0.791	*λ* = 0.356	*λ* = 0	*λ* = 0	*λ* = 0.975	*λ* = 0	*λ* = 0

*Note:* Significance of gray cells indicate independent variables in the model.

## 4 Discussion

We used a gradient of urban metal pollution to contrast two hypotheses explaining why species vary in the metal concentrations they can tolerate. We collected 26 species of butterflies that varied in concentrations of four metals that were elevated above levels of concern (lead, arsenic, cadmium, manganese). We found strong support for the range‐size‐tolerance hypothesis and weak support for the toxin coevolution hypothesis.

We found significant positive relationships between a species geographic range size and the concentrations of lead, cadmium, and arsenic in thoracic tissue (both maximum and predicted mean values, Table 2, Figures 3 and 4). This is consistent with other studies showing correlations between range size and tolerance to physiological stressors (e.g., Calosi et al. 2008; Gaston and Spicer 2001; Saetersdal and Birks 1997; rev. Bozinovic et al. 2011), and observations that butterflies that thrive in anthropogenic environments are also those with larger ranges (Clark et al. 2007; Jahner et al. 2011; Sunde et al. 2023). Species with larger ranges experience greater environmental variation, which should select for greater phenotypic plasticity that can accommodate survival in novel environments (Snell‐Rood and Ehlman 2021; Snell‐Rood et al. 2018). We broaden past work linking range size to thermal and urban tolerance to include metal tolerance. This finding has important implications for conservation—species with large ranges are more likely to succeed in polluted environments. Large range sizes are characteristic of invasive (or nonnative) species, as is their ability to tolerate a large range of conditions (Higgins and Richardson 2014), and their ability to tolerate pollution may lend to their continued success. Indeed, one nonnative species in our study ( *Pieris rapae* ) had not only one of the largest ranges, but also high concentrations of lead and arsenic, consistent with lab studies of lead tolerance in this species (Philips et al. 2017). Alternatively, species with small range sizes may be particularly affected by pollution (e.g., *Satyrodes eurydice* and *Lycaena dione* in this dataset), which could further their susceptibility to loss. We note that the common urban species we collected have a larger range size in general than more sensitive species (we did not sample species with particularly small range sizes).

We found only marginal support for the toxin coevolution hypothesis. Species with an evolutionary history with relatively more mutagenic host plant families had higher maximum levels of lead in their thoracic tissue (Table 2, Figure 3). However, there was no significant support for this hypothesis with respect to other metals or the average level of lead. Arguments from the pesticide literature suggest that insect species with evolutionary histories with plant defensive chemicals are more likely to evolve resistance to pesticides through the co‐option of underlying toxin processing (e.g., cytochrome P450s, oxidative stress responses; Li et al. 2007). We show potential support for this with lead, but not for general tolerance of metals. Our statistical power for this hypothesis was somewhat weaker as we had only 13 independent evolutionary host plant shifts represented in our dataset of 26 species. In addition, there were some plant families for which host plant mutagenicity data were deficient (e.g., Poaceae, Fagaceae, see Supporting Information). A sampling of more butterfly species across a range of cities would give more power, especially if other host plant associations were incorporated (e.g., the highly mutagenic Aristolochiaceae). Regardless, the lack of general tolerance gained from a co‐evolutionary history with mutagens suggests that novel pollutants may be inescapably harsh for most species; even after millennia of coevolution with mutagens, some species cannot cope with novel pollutants. Policy and industry need to address the impacts of pollutants by thoroughly testing the ecological impacts of novel chemicals, reducing the use of known harmful pollutants, and remediating existing pollutants, such as lead.

We focused on four heavy metals in this research, choosing those where soil values in the Twin Cities showed elevated levels relative to levels of health concern. Of these four metals, we saw relationships between our predictor variables and arsenic, lead, and cadmium, but not manganese. Manganese was the least elevated of these four metals, so it is possible it was at less stressful levels. We also saw tighter relationships between lead and our predictor variables relative to cadmium and arsenic (Table 2, Figures 3 and 4). This could potentially be an artifact of our sampling design, which aimed to sample along a gradient of lead pollution (as we had more extensive public health data for lead).

This research has several implications for conservation in the face of pollution. First, it provides support for the idea that species with broad ranges are generally of lower conservation concern (Field et al. 2009; Sherwin et al. 2013), although even the tolerant and wide‐ranging *Pieris rapae* is in decline (Wepprich et al. 2019) and species with wide ranges are declining at faster rates than rare species (van Klink et al. 2024). Species with relatively smaller ranges such as *Satyrodes eurydice* or *Lycaena dione* might be particularly deserving of increased conservation efforts, as they may have an evolutionary history with relatively lower environmental variation. Indeed, a recent study on widespread butterfly declines highlights > 70% cumulative declines in these two species over the last 20 years (Edwards et al. 2025). Second, this research provides some support that an evolutionary history with mutagenic host plants might provide some additional resilience. For example, grass‐feeding species may be somewhat more susceptible than those feeding on more mutagenic host plant families, such as Brassicaceae (Figures 2 and 3). Third, this research more generally points to the importance of reducing and remediating pollution and providing urban habitat for butterflies in areas of low pollution. Often, the pockets of available habitat for new conservation efforts are in areas with a legacy of pollution; we generally recommend continuing to restore these habitats. However, additional conservation efforts in areas of low pollution and the addition of soil amendments or plantings that reduce exposure to species in areas of high pollution will increase the chances that more sensitive species will also benefit from conservation efforts.

As a key component of our analyses, we assembled a large database of mutagenicity in plants that will likely be of use in future comparative studies. Although evolutionary history with plant mutagens was not particularly important in the present study, it could have had consequences for rates of mutation and evolution in the short term (Fordyce 2010) and selection for DNA repair and longer lifespans in the long‐term (Beck and Fiedler 2009; Promislow 1994). There are also interesting questions about why plant families vary in mutagenicity (e.g., Figure 2) that call for more sophisticated phylogenetic analyses and hypotheses.

This study's field approach allowed us to sample more species than would have been feasible to rear in the lab and to obtain results that are inherently relevant to natural conditions. However, such an approach comes with limitations. First, we used measures of metal concentrations of wild‐collected individuals to infer something about a species' tolerance, assuming that patterns of population‐level survival reflected tolerance and body metal concentration reflected exposure. Most studies on insects find that individuals reared on higher metal diets as larvae have a signature of higher tissue metal concentrations as adults (Ding et al. 2013; Reich et al. 2023), making insects good bioindicators of metal pollution (Pallottini et al. 2023). However, it is important to note that some studies in plants show that adaptations to elevated metals can result in greater metal excretion and lower individual concentrations of metals in metal‐adapted populations (Belimov et al. 2003; Meerts and Van Isacker 1997). We expect this is not the case for our study species given high gene flow across the urban‐to‐rural gradient for the species in this study that is unlikely to allow for local adaptation to metal pollution (Stevens et al. 2010). Regardless, lab studies of controlled metal concentrations and dose–response curves would ideally be needed to confirm species differences seen in this study, perhaps with a subset of species varying in larval hosts and range size. Studies with artificial diets containing zinc do corroborate some differences seen here; namely the butterfly *Pieris rapae* , which has a relatively high mutagenicity history, is relatively resistant to high levels of zinc, while *Danaus plexippus* , with a lower mutagenicity history, is much more sensitive to zinc (Shephard et al. 2020; note that *P. rapae* also has a larger range, but only by 6% relative to *D. plexippus* ).

A second limitation of the field approach is that it is correlational. It is possible that a factor other than metal concentrations could be the primary causal determinant of the observed patterns. For example, a different pollutant, correlated with heavy metal pollution, could be the primary causal factor. There are also other differences across these butterfly species that could potentially explain patterns of variation. For instance, host breadth could also select for tolerance of toxins through mechanisms similar to the toxin coevolution hypothesis (Ali and Agrawal 2012; Cornell and Hawkins 2003). Although our total number of species limited the number of independent variables we could include in our analyses, we performed post hoc analyses to test the importance of niche breadth, using total number of reported hosts (Scott 1992) as a measure of niche breadth. Comparing these models (Table S1) to our primary models (Table 2) generally showed no qualitative changes in the relationships between range size, mutagenicity, and metal loads. In general, the addition of host breadth did not significantly improve model fit, with the notable exception of predicted cadmium and maximum lead levels having marginally significant improved model fits (Table S1). Interestingly, the maximum lead model suggests joint effects of host breadth and mutagenicity, with mutagenicity having a more pronounced effect in species with higher niche breadth (Figure S4). This, coupled with the observation that for some metals there is a negative relationship between host breadth and metal loads (e.g., manganese) suggests more analyses are needed using a comparative dataset with greater power.

Why are some species able to tolerate novel pollutants? We present evidence that a species range size provides a broad tolerance to pollutants, likely from their experience with environmental variation, building on evidence that range size assists tolerance to temperature and urbanization. We also present weak evidence for a species' tolerance resulting from their coevolution with toxins. Pollution is a major cause of biodiversity loss; understanding species' susceptibility to pollutants advances our knowledge of species loss and informs targeted conservation of the most vulnerable species.

## Conflicts of Interest

The authors declare no conflicts of interest.

## Supporting information


Data S1

## Data Availability

All data are available online. The Ames test database and the species means used in the analyses are available through Mendeley (DOI: 10.17632/w42p9p7hpz.1). Heavy metal loads of butterflies are available through the Environmental Data Initiative (EDI; Kemmerling, Darst et al. 2025; Kemmerling, Snell‐Rood et al. 2025—DOI: https://doi.org/10.6073/pasta/368e1b0985fa750d3877107657b55cc4) and the Minneapolis‐St. Paul Long Term Ecological Research public online data catalog (https://mspurbanlter.umn.edu/datapublicationsmedia/data‐minneapolis‐st‐paul‐lter).
