# Supplementary material for: Geographic Range Size Predicts Butterfly Species' Tolerance to Heavy Metals More Than Evolutionary History With Toxic Larval Diets
Source: Evol Appl. 2025 May 26;18(5):e70114. doi: 10.1111/eva.70114 (PMC12104827; doi:10.1111/eva.70114)
Supplement: Supplementary file 1 — Data S1 [file EVA-18-e70114-s001.docx]

**Supplemental Material**

***Supplemental Figure 1.*** Distribution of individuals collected across collection sites (34 total) for butterfly species with at least ten replicate captures.
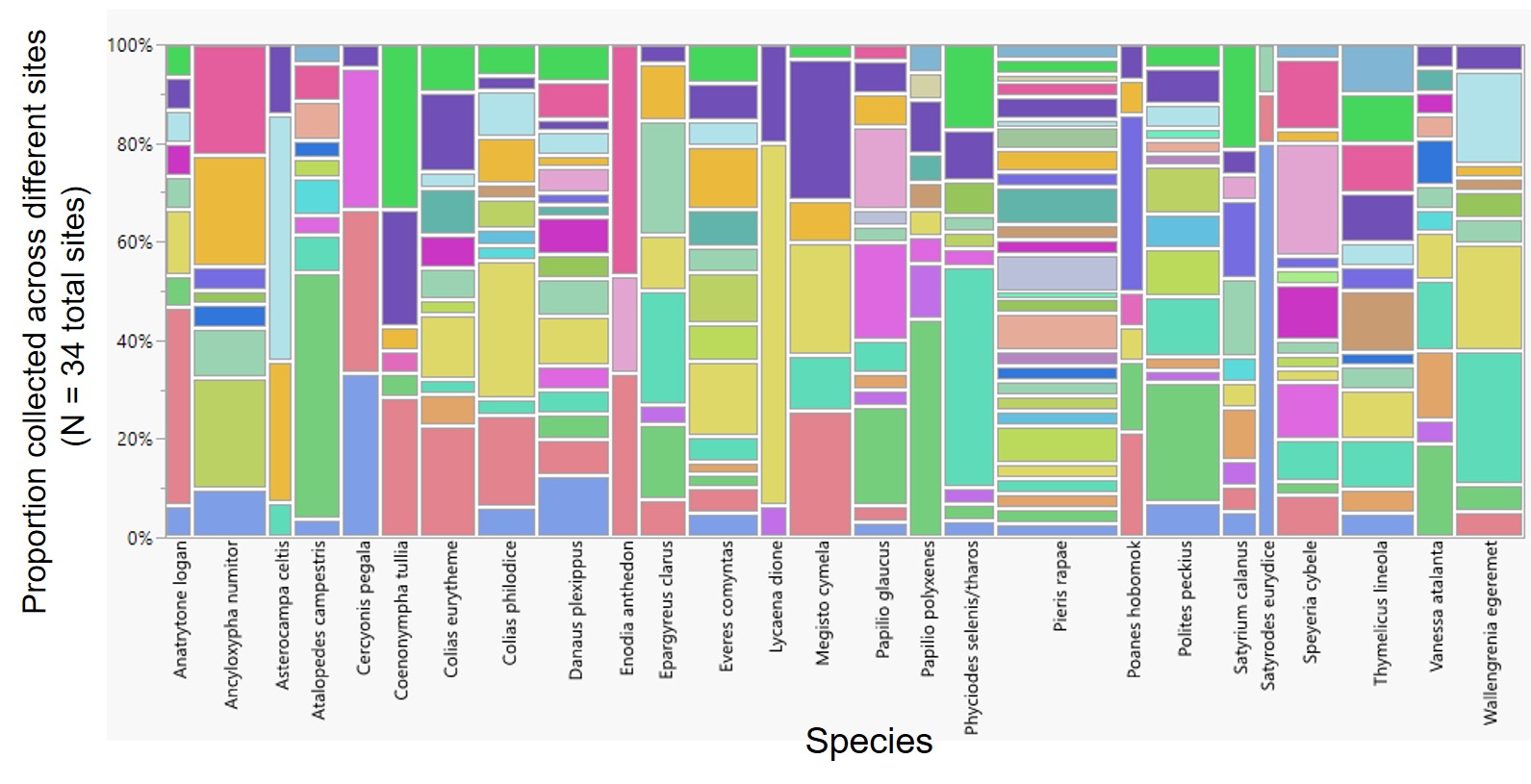


***Supplemental Figure 2.*** Smaller species showed a greater likelihood of samples below the detection limit for lead (Spearman’s rho = -0.67, *P* = 0.0002); this correlation was not significant for cadmium (Spearman’s rho = -0.24, *P* = 0.23), although the two species with the greatest samples below limit of detection were small species.

***
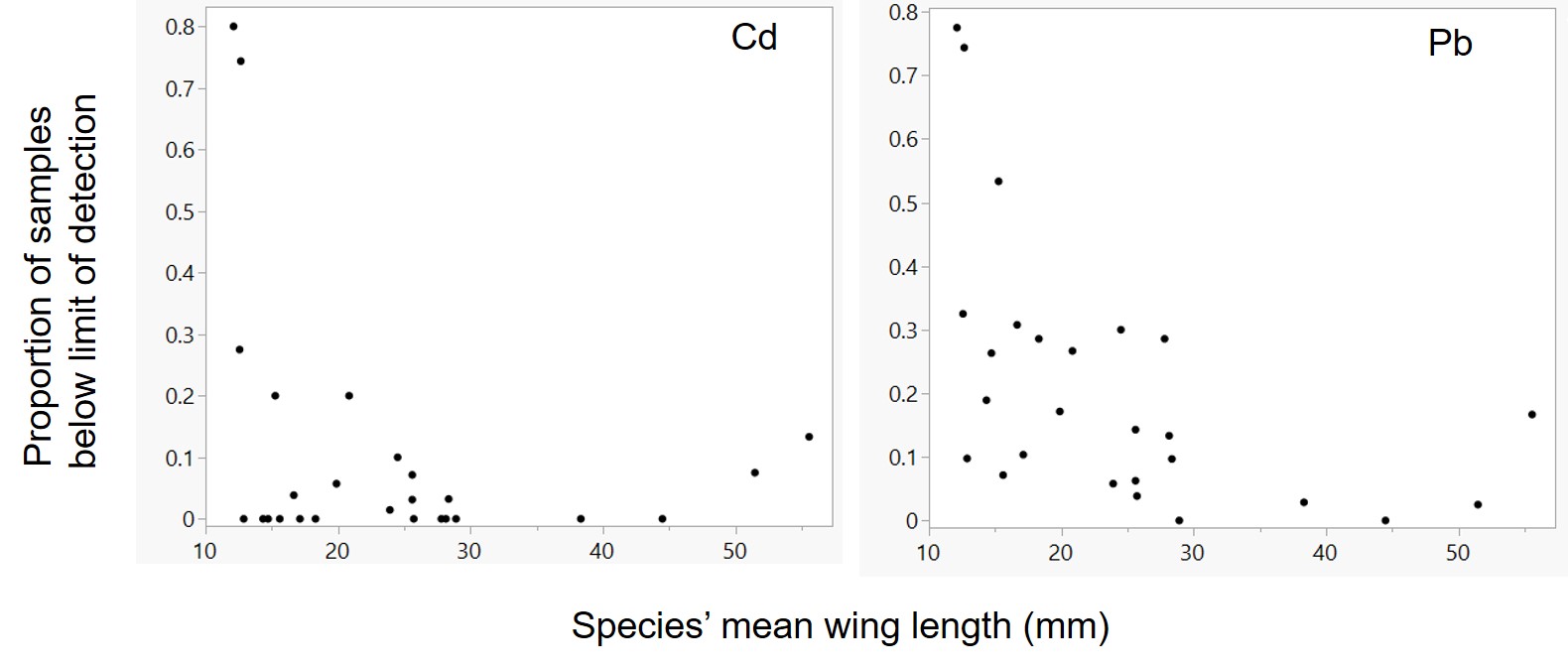
***

***Supplemental Figure 3.*** Shown are plots of raw data for thorax concentrations of manganese, cadmium, arsenic and lead. For each value above the limit-of-detection, we log-transformed concentrations (plus one). Samples below the limit-of-detection are plotted as zero and were accounted for in the analysis (see methods). Graphs are ordered by mean concentration, noting that the ranking changes for each metal. See Table 1 for statistics.

**
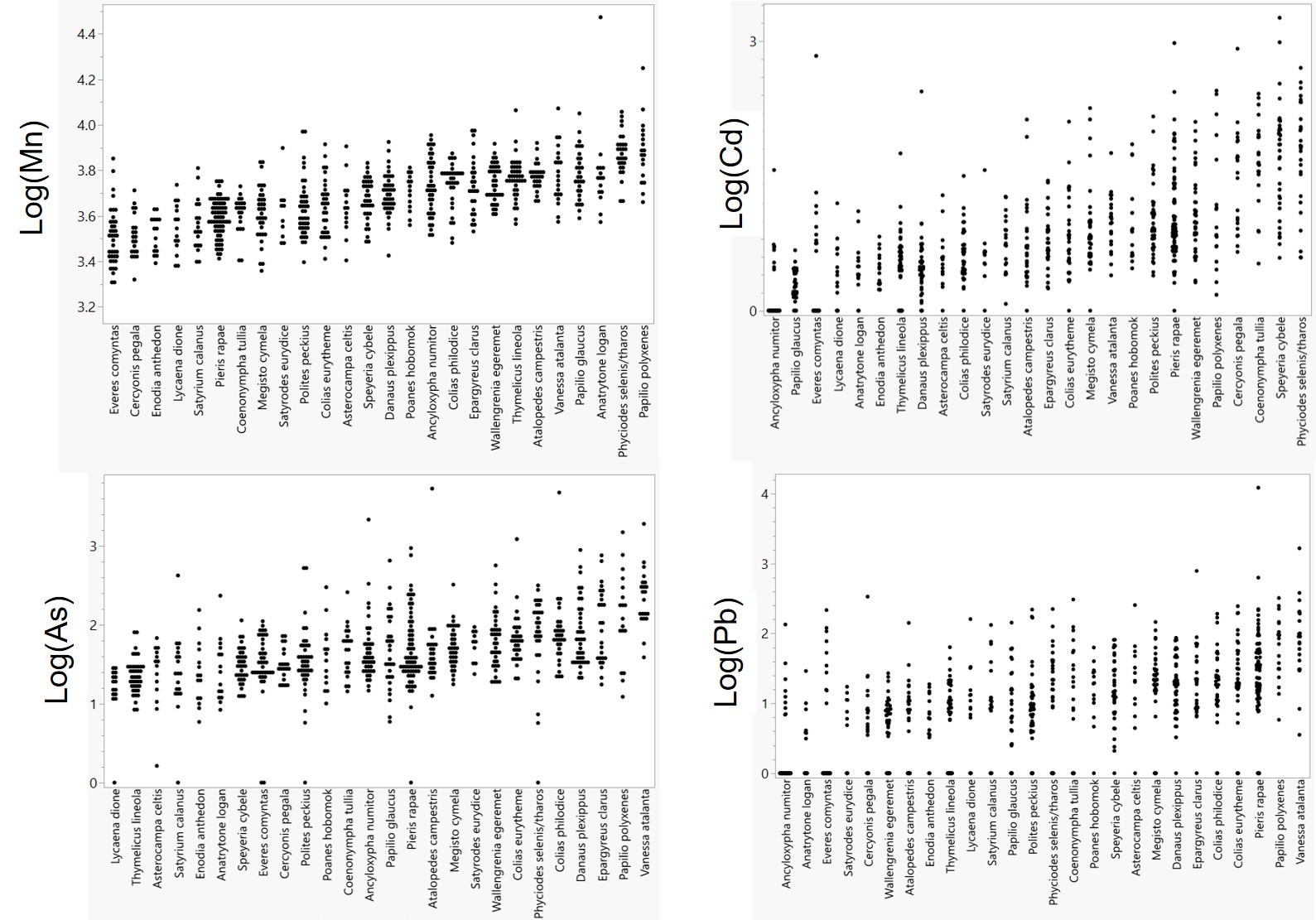
**

**Supplemental Figure 4.** Visualization of interaction between host breadth and mutagenicity seen for maximum lead levels (see Supplementary Table 1). For visualization purposes, we categorized a species’ host breadth as “high” (greater than or equal to the median number of host species) or “low” (less than the median number of host species).

**
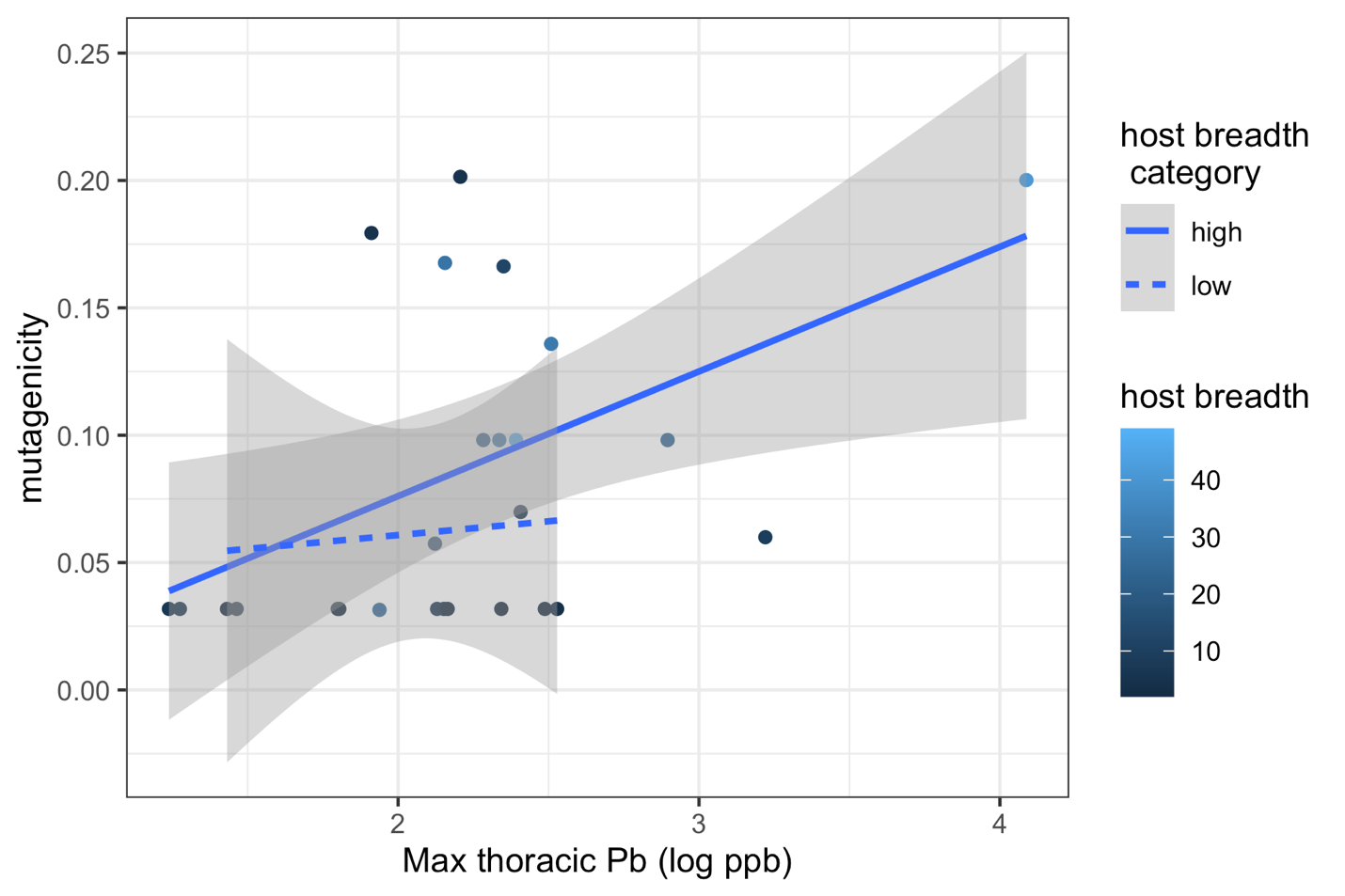
**

**Supplementary Table 1. Repetition of PGLS models with inclusion of host breadth.** Models reported in Table 2 were repeated with the inclusion of a species host breadth (number of species of hosts reported in Scott 1992). The bottom row shows the comparison between this model and the model without host breadth (Table 2).

|  | | Dependent variable (metal) | | | | | | | |
| --- | --- | --- | --- | --- | --- | --- | --- | --- | --- |
|  |  | Max(Mn) | Predicted(Mn) | Max(As) | Predicted(As) | Max(Cd) | Predicted(Cd) | Max(Pb) | Predicted(Pb) |
| Independent variables | Range Size | t = 0.46  *P* = 0.65  Est. = 0.02 | t = 1.20  *P* = 0.24  Est. = 0.03 | t = 1.93  *P* = 0.07  Est. = 0.22 | t = 2.16  *P* = 0.04  Est. = 0.11 | t = 2.50  *P* = 0.02  Est. = 0.37 | t = 3.71  *P* = 0.001  Est. = 0.32 | t = 3.37  *P* = 0.003  Est. = 0.31 | t = 2.25  *P* = 0.04  Est. = 0.22 |
|  | Mutagenicity | t = -0.12  *P* = 0.91  Est. = -0.005 | t = 0.75  *P* = 0.46  Est. = 0.02 | t = -1.07  *P* = 0.29  Est. = -0.13 | t = -0.66  *P* = 0.52  Est. = -0.04 | t = 0.76  *P* = 0.45  Est. = 0.12 | t = 2.01  *P* = 0.06  Est. = 0.19 | t = 2.33  *P* = 0.03  Est. = 0.23 | t = 1.30  *P* = 0.21  Est. = 0.14 |
|  | Breadth | t = 0.41  *P* = 0.69  Est. = 0.02 | t = -4.26  *P* = 0.0004  Est. = -0.05 | t = 1.33  *P* = 0.20  Est. = 0.19 | t = 1.10  *P* = 0.28  Est. = 0.07 | t = -0.96  *P* = 0.35  Est. = -0.18 | t = -3.06  *P* = 0.006  Est. = -0.34 | t = -0.64  *P* = 0.53  Est. = -0.07 | t = 0.04  *P* = 0.97  Est. = 0.005 |
|  | Mut:Breadth | t = -0.18  *P* = 0.86  Est. = -0.009 | t = -0.39  *P* = 0.70  Est. = -0.01 | t = 0.37  *P* = 0.72  Est. = 0.05 | t = 0.03  *P* = 0.97  Est. = 0.002 | t = 0.18  *P* = 0.86  Est. = 0.03 | t = 0.65  *P* = 0.52  Est. = 0.06 | t = 2.11  *P* = 0.047  Est. = 0.21 | t = 0.01  *P* = 0.99  Est. = 0.001 |
| Likelihood ratio | | LR = 0.21  *P* = 0.90 | LR = 1.02  *P* = 0.60 | LR = 2.73  *P* = 0.26 | LR = 1.75  *P* = 0.42 | LR = 1.18  *P* = 0.55 | LR = 5.70  *P* = 0.06 | LR = 5.07  *P* = 0.08 | LR = 0.004  *P* = 0.998 |

**Appendix S1. Constructing the Plant mutagenicity database**

***Building the database***. We used the existing literature to compile a database of mutagenicity across plant families. We focused on the standard “Ames test” screen, which has been in use since the early 1970s [1-3] and is a common method to test plant extracts for mutagenic compounds. There is, of course, a bias in the plants that researchers choose to screen, typically towards those used in traditional medicine or culinary applications; we discuss this bias further in the manuscript.

We used “Web of Science” to perform a broad literature search for Ames test data from plant extracts. We primarily used the search phrase ["Ames test*" and (plant* or vege* or herb*or leaf or leaves or root* or stem* or flower*)] to focus the search on various herbaceous plant parts. This search returned over 2000 papers (through March 2023), which we read through manually for appropriate papers. We chose papers that used Ames testing and some kind of plant extract (e.g., extract of leaves or roots). We avoided papers that were focused on a chemical that had been identified in a plant, but was tested as an individual chemical (e.g., “compound A which was discovered in plant X”). For a subset of plant families that butterflies feed on, the Ames test sampling was very poor (e.g., Poaceae), so we ran additional searches that included the genera of those species fed on by the butterflies in the study (within Poaceae, Fagaceae, Violaceae, Betulaceae, Polygonaceae, Salicaceae, Cyperaceae). For instance, the search for grasses was: ["Ames test*" and (Poaceae or Gramineae or grass* or Poa or Cynodon* or bamboo* or pasture* or cereal* or corn* or maize* or wheat* or Triticum or rye* or Triticeae or oat* or Avena or barley* or Hordeum)].

In total, our database included data from 163 studies (from 1983-2023), including 502 species of 103 plant families within 37 plant orders. For each study, we read through the abstract, methods and results and took the following data. First, we focused on “was the plant sample found to be mutagenic” as our primary response variable, with a “yes-no” category as the focal variable. This categorization was the most easily standardized metric across studies as the standard curves and details of the tests varied with studies. Second, we took notes on several key aspects of the methodology for a study, including the solvent used, whether the assay was run on an agar plate (the classic method) or on a 96-well plate (more recent students), the number, species and strains of bacteria used in the assay, the part of the plant where the tissue extract originated, and whether or not an activating enzyme was used in the assay. We discuss the distribution and effects of this methodological variation below.

We first asked about the agreement between tests run on the same extract, but with or without an activating enzyme. The majority of such paired tests agreed the sample was not mutagenic (Table 1). However, for tests that detected a positive Ames test result, 46% detected a positive test with the activating enzyme (but not without the enzyme), and 20% detected a positive test without the activating enzyme, but not with. This variation presents challenges for merging studies with replicate enzymatic tests (820 tests) with those with only one test (205 additional tests). To combine findings across studies and tests, in the subsequent analyses, we treat “with” and “without activating enzyme” separately, but avoid pseudoreplication in our final calculations of plant family mutagenicity by including “species” as a random effect. This approach is broadly consistent with our interest in estimating the “probability” that a given plant extract is mutagenic to an herbivore, without necessarily making assumptions about the digestive physiology of that animal (e.g., species vary in their ability to detoxify certain chemicals).

**Table 1.** ***Agreement between tests with and without activating enzymes***. Across all studies in our starting database, there were 820 tests that ran the Ames test assay on an extract with both the enzyme activation, and without (there were an additional 205 tests that ran the test only without the activating enzyme).

|  | Without enzyme negative | Without enzyme positive |
| --- | --- | --- |
| With enzyme negative | 645 | 35 |
| With enzyme positive | 80 | 60 |

***Variation in methodology***. We tested whether variation in Ames test methodology across studies resulted in variation in the probability of detecting whether an extract was mutagenic. As many of the factors were highly correlated, we focused on individual chi-square tests for parameter estimates as a full model produced unstable parameter estimates. However, we use model selection to determine which factors to correct for when calculating plant family means (see below, Table 2). In these analyses, we treat each test (with or without activating enzyme) as an observation, but given that nominal logistic models are not capable of random effects (at least in JMP v17), it is important to note that reported *P* values are thus somewhat inflated.

First, we considered the effect of solvent: across the 163 studies, there were 28 solvent categories used, the most common being methanol (22%), DCM (19%), ethanol (14%), and water (16%). For the ten solvent types with at least 20 replicates, type had a significant effect on the probability of detecting mutagenicity (N = 1593 tests, X^2^ = 120.7, P < 0.0001) with the highest probability for PBS (41% yes-mutagenic) and water (22%) and the lowest for petroleum ether (0%) and DCM (3.25%). Given that we had 28 total solvent categories, we decided to focus further analysis on two solvent characteristics that likely affected chemical extraction: whether the solvent was organic (or inorganic) and its polarity. Non-organic solvents were significantly more likely to result in a positive Ames test (25% of the time) than organic solvents (10% of the time; N = 1695, X^2^ = 48.6, P < 0.0001). In addition, polar solvents were significantly more likely to result in a positive test (16%) than nonpolar (0%) or slightly polar (3.25) solvents (N = 1695, X^2^ = 65.1, P < 0.0001).

Second, we asked how characteristics of the bacteria affected the probability of a positive Ames test. Across the 163 studies, there were 26 strains of bacteria used, from two species (98% *Salmonella typhimurium*, <1% *Escherichia coli*, *Gymnema sylvestre* or mixture of species). The most common strains of *Salmonella* used were TA98 (42.9% of tests) and TA100 (36.5% of tests). Given the large number of strains used, we focused on whether the number of strains used affected the probability of a positive tests result. As the number of strains increased, the probability of a positive test result also increased, from 1.85% with one strain to 27.4% with four or more strains (N = 1765, X^2^ = 67.6, P < 0.0001). We additionally asked whether the agar plate versus the 96-well plate method (also known as the fluctuation method) would matter, and, unexpectedly, the probability of a positive test result was lower with the agar plate method (12.3%) than the 96-well plate method (30.2%, N = 1579, X^2^ = 30.6, P < 0.0001), possibly because replicate rate could be higher on a 96-well plate.

Third, we asked whether the presence of an activating enzyme (generally a rat liver enzyme) made a difference in detecting mutagenicity (for distribution of tests, see Table 1). There was no effect of enzyme activation on the probability of a positive test (N = 1765, X^2^ = 0.45, P = 0.51).

Finally, we considered the part of the plant used in the extract. Most of our samples were either leaves, or whole plants (59% of samples), as we focused our search on leaves, the primary plant food source for butterfly larvae. A smaller proportion of samples came from roots or bulbs (16%) and stem or bark (15%) and flowers, fruit or seeds (9%). In the chi-square test focusing solely on this factor, plant part had a significant effect on the probability of a positive test (N = 1755, X^2^ = 17.4, P = 0.0006) with the highest probability for flowers, fruit, and seeds (17.5%), followed by leaves and whole plants (15.3%), and the lowest for stem/bark (7.6%) and roots or bulbs (9.9%).

To compare the relative importance of all factors together, we constructed a single logistic model to test for all factors together. A model selection approach focused on the log-worth of each factor resulted in the elimination of “activating enzyme” and “plant part” from the full model. This model suggests that whether the solvent is polar, the use of the agar plate or fluctuation methods, and the number of strains, may be particularly important in the probability of detecting a positive test (Table 2). That said, several estimates in the model were unstable given the number of categorical factors and their distribution (likely due to collinearity), supporting the use of individual tests to estimate parameter effects.

**Table 2. Results of nominal logistic model testing the importance of methodological variation** for the 1507 tests with full methodological details. Full model is shown on the left, model following model selection on the right (terms with log-worth of 0.29, 0.32).

|  | Full model | | After model selection | |
| --- | --- | --- | --- | --- |
| Factor | X^2^ | *P* | X^2^ | *P* |
| Organic solvent | 11.0 | 0.0009 | 11.0 | 0.0009 |
| Polar solvent | 21.2 | <0.0001 | 22.1 | <0.0001 |
| Plate or well | 20.1 | <0.0001 | 24.4 | <0.0001 |
| Part category | 2.5 | 0.47 |  |  |
| Number strains | 25.3 | <0.0001 | 24.5 | <0.0001 |
| Activating enzyme? | 0.42 | 0.52 |  |  |

***Estimating plant family means.*** We were interested in estimating the average likelihood that a plant from a given family would be mutagenic. However, given the importance of variation in methodology discussed above, we wanted to correct for methods in merging the findings across studies. Because many of the factors are collinear, it is not possible to build a single model and get stable parameter estimates for each plant family. Thus, we adopted the following approach. For each sample, we calculated the probability of detecting a positive test result given a certain methodology that had a significant effect described above (solvent polarity, solvent organic, plant part, number of strains used, plate/well method). If there was information missing from the study on that particular methodological detail (13% of the time), we used the global probability of a positive result (13.428). Then we calculated the average likelihood that a test result would be positive based on the methods used. For instance, to calculate the probability of a positive test for an extract of leaves in acetone with 2 strains of bacteria on an agar plate: take the average of 13.2% (strains), 12.4% (agar plate), 15.3% (leaves), 10% (organic solvent), and 16.2% (polar solvent) = 13.4% probability (i.e., 0.134) of detecting a positive result. We then compared this value to the global probability of a positive result (13.428) to estimate whether a method increased or decreased the likelihood of a positive result. To do so, we subtracted this value from the observed value, where zero was a negative result and 1 was a positive result. In the previous example, with a negative result (0), the adjusted value was (0 – (13.43-13.428)/100) = -0.00002). This method results in an increased weighing for a positive test result when the methodology predicted a negative result, and a decreased weighting for a positive test result when the methodology predicted a positive test result.

To calculate the probability that an extract of a plant family is mutagenic, we took the family-level estimates for a model predicting each Ames test result, adjusted for methodology (see above), with “species” as a random effect to control for the fact that some tests were duplicated for individual species. In such a model, “Family” is a significant predictor of the probability that a plant extract is mutagenic (*F_56,283_* = 1.46, *P* = 0.024 for 56 families with at least 8 replicates, total N = 1610). These family-level means are highly correlated with a simple measure of mutagenicity that measures the proportion Ames tests that are positive, without correcting for any methodological detail (Spearman’s rho = 0.959, P < 0.0001). We also calculated order-level values for all tests run to substitute in cases where the family-level did not meet the minimum sample size of eight. Interestingly, there were no order-level significant effects in a model treating “species” as a random effect (N = 1761, *F_36,386_*=1.07, *P* = 0.36)


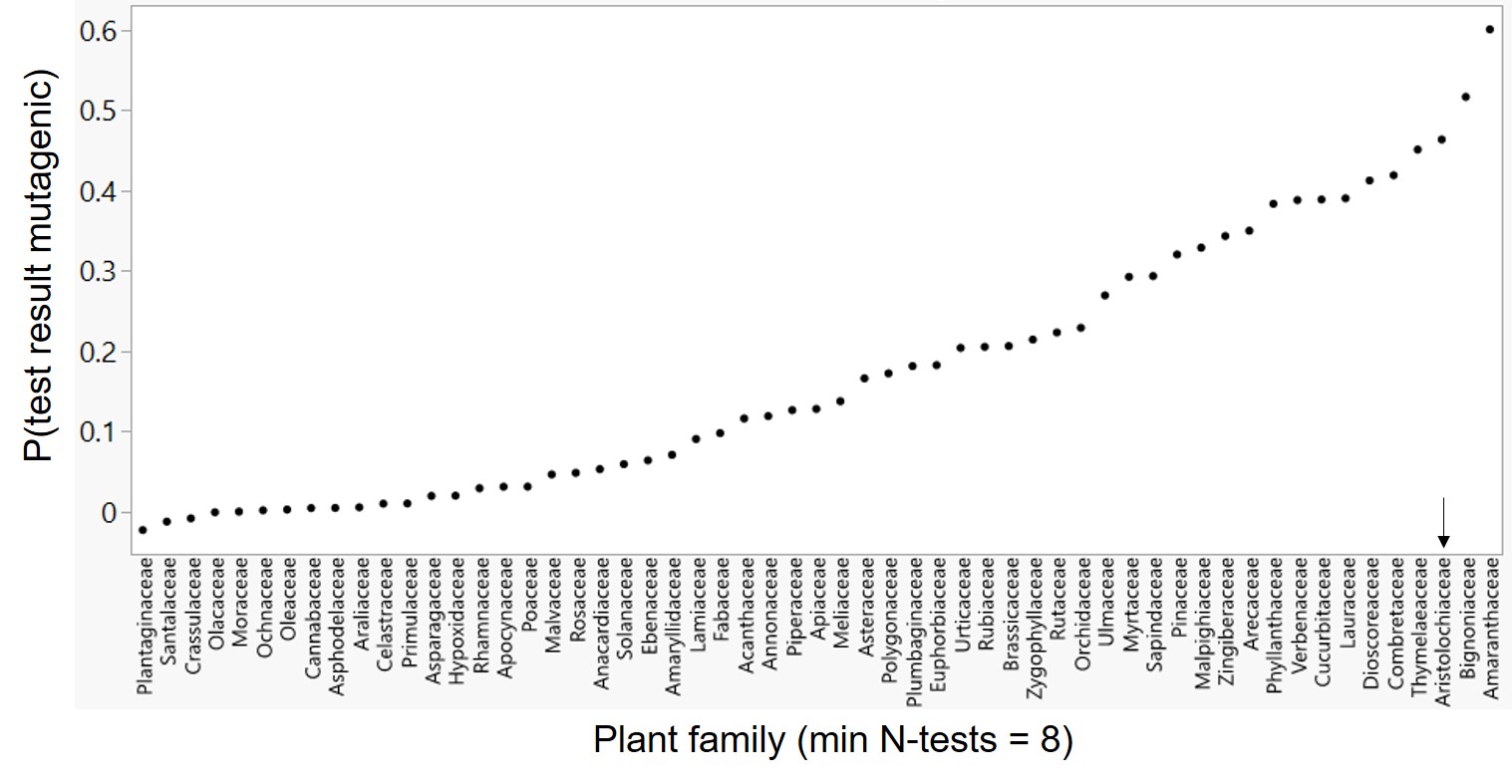


***Figure 1. Plant family means.*** The y-axis denotes the probability an extract from a given plant family will be mutagenic with an Ames test. These values are limited to families where at least eight tests were present in our full dataset. Values are corrected for variation in methodology and come from a model that treats “species” as a random effect. As proof of principle, the arrow indicates one plant family (Aristolochiaceae) with known mutagenicity [4-7].

**Literature Cited**

1. Ames, B.N., F.D. Lee, and W.E. Durston, *IMPROVED BACTERIAL TEST SYSTEM FOR DETECTION AND CLASSIFICATION OF MUTAGENS AND CARCINOGENS.* Proceedings of the National Academy of Sciences of the United States of America, 1973. **70**(3): p. 782-786.

2. Ames, B.N., *DIETARY CARCINOGENS AND ANTICARCINOGENS - OXYGEN RADICALS AND DEGENERATIVE DISEASES.* Science, 1983. **221**(4617): p. 1256-1264.

3. Ames, B.N., R. Magaw, and L.S. Gold, *RANKING POSSIBLE CARCINOGENIC HAZARDS.* Science, 1987. **236**(4799): p. 271-280.

4. Arlt, V.M., M. Stiborova, and H.H. Schmeiser, *Aristolochic acid as a probable human cancer hazard in herbal remedies: a review.* Mutagenesis, 2002. **17**(4): p. 265-277.

5. Chen, T., *Genotoxicity of aristolochic acid: a review.* Journal of Food and Drug Analysis, 2007. **15**(4): p. 10.

6. Frei, H., et al., *ARISTOLOCHIC ACID IS MUTAGENIC AND RECOMBINOGENIC IN DROSOPHILA GENOTOXICITY TESTS.* Archives of Toxicology, 1985. **56**(3): p. 158-166.

7. Schmeiser, H.H., B.L. Pool, and M. Wiessler, *MUTAGENICITY OF THE 2 MAIN COMPONENTS OF COMMERCIALLY AVAILABLE CARCINOGENIC ARISTOLOCHIC ACID IN SALMONELLA-TYPHIMURIUM.* Cancer Letters, 1984. **23**(1): p. 97-101.
